# The use of a novel signal analysis to identify the origin of idiopathic right ventricular outflow tract ventricular tachycardia during sinus rhythm: Simultaneous amplitude frequency electrogram transformation mapping

**DOI:** 10.1371/journal.pone.0173189

**Published:** 2017-03-10

**Authors:** Abigail Louise D. Te, Satoshi Higa, Fa-Po Chung, Chin-Yu Lin, Men-Tzung Lo, Che-An Liu, Chen Lin, Yi-Chung Chang, Shih-Lin Chang, Li-Wei Lo, Yu-Feng Hu, Ta-Chuan Tuan, Tze-Fan Chao, Jonan Liao, Yao-Ting Chang, Chung-Hsing Lin, Yuan Hung, Shinya Yamada, Kuo-Li Pan, Yenn-Jiang Lin, Shih-Ann Chen

**Affiliations:** 1 Division of Cardiology, Department of Medicine, Taipei Veterans General Hospital, Taipei, Taiwan; 2 Cardiac Electrophysiology and Pacing Laboratory, Division of Cardiovascular Medicine, Makiminato Central Hospital, Okinawa, Japan; 3 Faculty of Medicine and Institute of Clinical Medicine, National Yang-Ming University, Taipei, Taiwan; 4 Research Center for Adaptive Data Analysis and Center for Dynamical Biomarkers and Translational Medicine, National Central University, Jhongli, Taiwan; University of Minnesota, UNITED STATES

## Abstract

**Introduction:**

The signal characteristics of intracardiac bipolar electrograms at the origin of idiopathic RVOT-VT during sinus rhythm remain unclear.

**Objective:**

The study sought to develop a novel real-time/online technique, simultaneous amplitude frequency electrogram transformation (SAFE-T), to quantify and localize the diseased ventricular substrate in idiopathic RVOT-VT.

**Methods:**

We retrospectively investigated the intracardiac bipolar recordings in 70 consecutive patients (26% male, mean age 42±12 years) who underwent successful radiofrequency catheter ablation of idiopathic RVOT-VT. We quantified the extent of the frequency fraction of ventricular potentials during sinus rhythm or ventricular pacing using a novel formula, the product of instantaneous amplitude and frequency, and showed that in a 3D geometry as an online SAFE-T map.

**Results:**

The characteristics of the HHT spectra of electrograms derived from VT origins demonstrated high frequency components (>70 Hz), which were independent of the rhythm. The density of the abnormal potentials at the VT origins were higher (VT origins, 7.5±2.3 sites/cm^2^ vs. surrounding myocardium, 1.5±1.3 sites/cm^2^, *p*<0.001), and were significantly decreased after ablation (0.7±0.6 sites/cm^2^, *p*<0.001). A small region of abnormal potentials were observed in the VT origins (mean area of 1.5±0.8 cm^2^). The SAFE-T maps predicted the VT origins with 92% sensitivity, 78% specificity with optimal cut-off value of >3.0 Hz·mV.

**Conclusion:**

The online SAFE-T map was feasible for quantifying the diseased ventricular substrate, irrespective of the rhythm of activation, and can be used to identify the optimal ablation targets for idiopathic RVOT-VT. We found a limited region of abnormal potentials where the RVOT-VT origins were successfully ablated.

## Introduction

The majority of idiopathic ventricular arrhythmias (VAs) originate from the right ventricular outflow tract (RVOT). The mechanism of outflow tract VAs has previously been reported as focal.[1–6] Radiofrequency energy applications to the earliest depolarization site during VAs can cure this type of arrhythmia.[6–9] However, conventional mapping can be technically challenging in case with infrequent or noninducible VAs during the procedure. Recent studies have reported a novel ablation strategy without any induction of VTs targeting the local abnormal ventricular activity (LAVA) during sinus rhythm in structural heart disease.[10,11] Furthermore, the total elimination of LAVAs during sinus rhythm as an endpoint of catheter ablation of organic VTs have been proven to improve the arrhythmia-free survival of these patients.[12,13] Currently, LAVAs were identified by a subjective visual inspection manner and an automatic algorithm to identify and quantify the density of LAVAs is still not available. Furthermore, the signal characteristics of the intracardiac bipolar electrograms at the origin of RVOT-VT during sinus rhythm remain unclear. The Hilbert Huang Transform (HHT) analysis has been reported as a novel signal processing algorithm for nonlinear and nonstationary signals.[14,15] Thus, the aim of this study was to develop a novel real-time/online HHT with a temporal frequency analysis to quantify and localize the diseased ventricular substrate during sinus rhythm or right ventricular pacing and investigate the efficacy of this novel mapping method.

## Methods

### Study population

A total of 70 consecutive patients who underwent a detailed electrophysiological study (EPS) and successful radiofrequency catheter ablation (RFCA) of idiopathic RVOT-VT guided by 3D electroanatomical mapping were enrolled. All patients received 12-lead resting ECG, 24-hour Holter monitoring, 2D echocardiography, left and right ventriculography, and coronary arteriography prior to or during the index ablation procedure to exclude the possibility of coronary artery disease and any structural heart disease. Available cardiac magnetic resonance imaging (MRI) studies from the population were also reviewed to rule out structural heart disease. Baseline characteristics were assessed in detail.

### Electrophysiological study and electroanatomic mapping

This study was an observational study using online analysis utilizing the user-defined map module in CARTO system, MEM version 3.2 (Biosense Webster Inc., CA) that was conducted at and approved by the institutional review board of Taipei Veterans General Hospital, Taiwan. After written informed consent was obtained, a standard EPS protocol was performed in the fasting and non-sedated state. Before the study, all antiarrhythmic drugs were discontinued for at least 5 half-lives. If there was no spontaneous onset of VAs at baseline, the programmed ventricular stimulation protocol with or without intravenous isoproterenol infusion (1–5 μg/min) was performed as previously described.[6,7,12–14] The 3D electroanatomic (EAM) geometry, voltage and activation maps +/- pace maps were created using CARTO 3 system, and a 4-mm tip non-irrigated or 3.5-mm tip open-irrigated catheter (NaviStar or NaviStar ThermoCool, Biosense Webster), which contained a 2-mm ring electrode with a 1-mm interelectrode distance. Bipolar electrograms were sampled at 1 kHz and filtered at 30-500Hz and unipolar electrograms were filtered at 2-240Hz. To avoid low voltage recordings due to poor contact, we used the criteria previously described by Lin CY et al.[15] Activation mapping, defining the earliest local electrical signals, with or without a pace mapping technique, that compares the 12-lead QRS morphology during pace mapping with the clinically documented PVCs/VTs aiming for at least 11 of 12 leads matched, was performed. The target ablation site was selected on the basis of the earliest activation site and/or the site of the optimal pace mapping.

### Catheter ablation and follow-up

RF energy was applied in a temperature-controlled mode (<50°C) with a pulse duration of 60 seconds; maximum power of 50W for non-irrigated catheter and 30-35W for irrigated catheter, and targeting for an impedance drop of 10Ω. If VAs were suppressed within 30 seconds, the RF application was continued for a total of 60 seconds and additional RF energy was applied for up to a maximum of 5 ablation lesions. Acute procedural success was defined as the complete elimination of spontaneous or inducible VAs under an isoproterenol infusion, following the same induction protocol for 30 minutes to exclude any acute recurrences. The VT origin was localized by an ablation site with acute success, and the VT origins were categorized into the RVOT septum, RVOT free wall, and RV body. After hospital discharge, the patients were followed up closely (every 1 to 3 months) in the cardiology outpatient clinic. The long-term efficacy was assessed clinically on the basis of the 12-lead ECGs, 24-hour Holter monitoring, and clinical symptoms. Recurrence was defined as recurrence of sustained VT, nonsustained VT, or greater than 1000 ventricular PVCs, as confirmed by the morphology criteria using 24-hour Holter monitoring.[16–18]

### Signal acquisition and Simultaneous Amplitude Frequency Electrogram Transformation (SAFE-T) mapping

**Fig 1:**
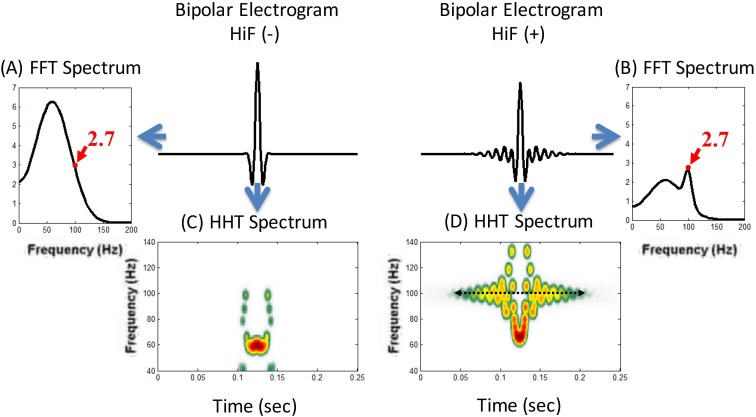
**The schematic comparison between the Fourier-based high frequency power spectra (A and B) and HHT spectra (C and D)**. Ambiguity of the Fourier-based high frequency power spectra between waveform without and with high frequency activity due to a window broadening effect while the corresponding HHT spectra can clearly identify high frequency component (> 70 Hz). FFT: fast Fourier transformation; HiF (-): bipolar electrogram without high frequency activity; HiF (+): bipolar electrogram with high frequency activity.

Here we proposed an automated method to recognize the abnormal high frequency potentials in sinus rhythm during ablation of idiopathic RVOT VT. Because the high frequency fractionated signals exhibit a highly nonlinear and nonstationary pattern and appeared intermittently, the traditional Fourier filter could not easily separate these highly fractionated components from the ventricular electrogram. The present method utilized a novel signal processing tool for nonlinear and nonstationary signals, the Hilbert Huang Transform (HHT).[14,15] **Fig 1** demonstrates a schematic comparison between the Fourier-based high frequency power spectra (A and B) and HHT spectra (C and D). The Fourier-based high frequency power spectra could not differentiate the bipolar electrogram waveforms without high frequency components (Fig 1A) from those with high frequency components (Fig 1B) due to the window broadening effect, thus, both registering a frequency at 100Hz. However, with Hilbert-Huang Transformation (HHT), the waveforms without high frequency components (Fig 1C) demonstrated a low frequency waveform at <70Hz while those with high frequency components (Fig 1D) registered a high frequency waveform at 100Hz. HHT was able to identify the high frequency components from the local ventricular electrogram which the Fourier-based analysis failed to demonstrate.

In this study, the bipolar electrograms from sinus rhythm voltage maps and activation/pace maps were exported and analyzed using HHT. HHT was used to derive the instantaneous amplitude and instantaneous frequency for each electrogram. The Simultaneous Amplitude Frequency Electrogram Transformation (SAFE-T) value is the product of the instantaneous amplitude and instantaneous frequency derived from the HHT, expressed as Hz·mV.[15] The SAFE-T value was used to quantify the high frequency fractionated components in the bipolar electrograms. The details of the the derivation of the instantaneous amplitude and instantaneous frequency from HHT and computation of the SAFE-T value can be found in S1 File and S1 Fig.

After analysis, the data set is imported into the CARTO system using the user-defined map (UDM) module and applied to the color-coded 3D electroanatomic map, called the SAFE-T map, for anatomic co-registration. Each point in the SAFE-T map registers a specific SAFE-T value (Hz·mV). We compared the mean SAFE-T values between the VT origin sites and the surrounding myocardium. The density of the abnormal high frequency fractionated signals was also computed. Here, we defined density as the number of sites with abnormal high frequency fractionated electrograms per square centimeters (sites/cm^2^).

The application for SAFE-T mapping for VT in structural heart disease has been previously described in detail.[15]

### Statistical analysis

All continuous variables were expressed as the mean ± SD. Categorical variables were presented as proportions and percentages. A receiver operating characteristic (ROC) curve analysis was applied to evaluate the sensitivity and specificity of the analysis, with the aim of predicting the optimal cut-off SAFE-T value. The predictive performance of the SAFE-T maps was evaluated using the Pearson’s correlation coefficient. The internal consistency of SAFE-T values between sinus rhythm and right ventricular pacing were evaluated by Cronbach’s α coefficient. A Cronbach’s α coefficient of 0.7 or more was considered satisfactory.[19] All statistical analyses were performed using commercial statistical SPSS software (version 20.0, IBM, New York).

## Results

### Patient characteristics

The patient population consisted of 70 consecutive patients, 18 (26%) were male, mean age of 42±12 years old, without any structural heart disease, who were referred to our institution for RFCA of idiopathic RVOT-VT. Tables 1 and 2 shows the clinical characteristics and electrophysiologic characteristics of the patients, respectively. All patients had a mean PVC/24 hours of 14,596±12,402 beats (VPC burden 16±11%); PVCs only in 42 (60%), sustained and nonsustained VT in 6 (8.6%) and 28 (40%), respectively, as recorded by 24-hour Holter monitoring. Coexistent atrial arrhythmias were noted in 5 patients (7.1%) which occurred prior to or after the index procedure for idiopathic RVOT-VT, and ablated on separate occasions.

**Table 1:** Clinical characteristics.

Variables	N = 70
Age (years, mean±SD)	42±12
Gender (male, n, %)	18 (26)
**Symptoms**	
Palpitations (n, %)	58 (83)
Dyspnea/Shortness of Breath (n, %)	10 (14)
Near-syncope/syncope (n, %)	5 (7)
**Clinical History**	
Hypertension (n, %)	8 (11)
Diabetes (n, %)	3 (4)
**Echocardiographic Parameters**	
LVEF (%, mean±SD)	60±9
Cardiac MRI Parameters*	
RVEF (%, mean±SD)	50±11
Delayed Hyperenhancement (n, %)	0 (0)
Structural and Wall motion Abnormality (n, %)	0 (0)
**24-hour Holter**	
PVC/24h (mean ± SD)	14,596±12,402
PVC burden (%, mean ± SD)	16±11
PVC only (n, %)	42 (60)
Nonsustained VT (n, %)	28 (40)
Sustained VT (n, %)	6 (8.6)
Coexisting Atrial Arrhythmia (n, %)	5 (7.1)
**Post-ablation Follow-up**	
Follow-up period (months, mean ± SD)	12±10
Isolated PVC recurrences (n, %)	10 (14.3)
Nonsustained VT recurrence (n, %)	0 (0)
Sustained VT recurrence (n, %)	0 (0)

* Cardiac MRI parameters were obtained from the available data in 52 patients in this study.

**Table 2:** Electrophysiologic characteristics.

Variables	N = 70
Induced PVC only (n, %)	50 (71.4)
Induced Sustained VT (n, %)	4 (5.7)
Induced Nonsustained VT (n, %)	15 (21.4)
Activation mapping (n, %)	53 (75.7)
Earliest activation time (msec, mean±SD)	32±19
Pace mapping (n, %)	68 (97.1)
RF energy applications (mean±SD)	11±6

### Catheter ablation and follow-up

During the EPS, sustained VT was induced in 4 (5.7%) patients, nonsustained VT in 15 (21.4%), and only PVCs were induced in 50 (71.4%) (Table 2). One patient had no inducible VT or PVC, and localization of the origin was done by pace mapping guided by the morphology from the clinically documented PVC. The mean procedure time was 37±26 minutes with 24,718 total mapping points (mean mapping points: 353±153 per patient), and the mean fluoroscopy time was 23±17 minutes. Activation mapping was performed in 53 (75.7%) patients, while pace mapping was performed in 68 (97.1%) patients. The mean earliest activation time before the onset of the QRS complex during the VAs at the successful ablation site was 32±19 msec. RF energy applications (11±6) at those sites successfully eliminated the PVCs/VTs in all cases and were not inducible by programmed electrical stimulation protocol under intravenous infusion of isoproterenol. During the follow-up period (mean follow-up: 12±10 months), 10 (14.3%) patients developed isolated PVC recurrence (>1000 beats/day). The QRS complex morphology of recurrent PVCs was similar to that before the RFCA in 9 out of the 10 patients with recurrence. There was no recurrence of the sustained or nonsustained VT during the follow-up period. Therefore, no patients in this study population underwent a second procedure.

### Signal characteristics of the substrate at the VT origin

A total of 4300 electrograms taken during sinus rhythm were analyzed and 84 RVOT-VT origins were identified: 65% originated from the RVOT septum, 25% from the RVOT free wall, and 10% from the RV body. At the VT origins, we found a limited region of abnormal potentials with a mean area of 1.5±0.87cm^2^ (0.83±0.52%).

**Fig 2:**
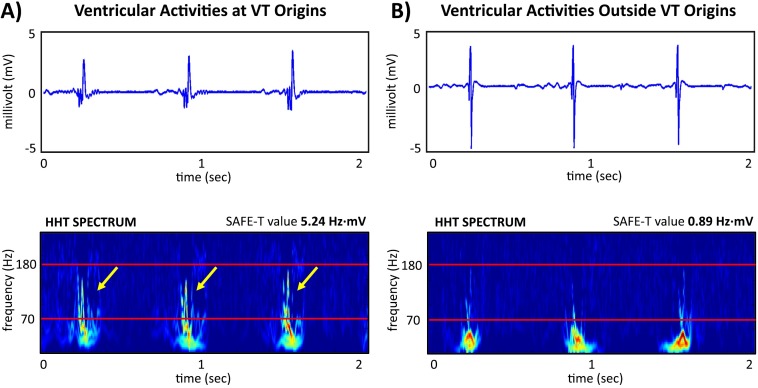
**Representative examples of high frequency activity of the bipolar electrogram at the VT origin (A) and surrounding regions (B)**. The corresponding temporal frequency HHT spectra demonstrates a heterogeneous shift toward high frequency components (>70 Hz) only at the VT origin (arrows, left lower panel) but not the surrounding area (right lower panel).

The characteristics of the HHT spectra of the bipolar electrograms derived from the VT origins during sinus rhythm demonstrated high frequency components (>70 Hz), which were independent of the rhythms (Cronbach’s α coefficient = 0.897) and highly correlated between different rhythms (Pearson’s coefficient (r) = 0.918, p<0.001). **Fig 2** demonstrated a representative example of the high-frequency activity (>70Hz) of a bipolar electrogram and the corresponding heterogeneous HHT spectra at the VT origin (Fig 2A), whereas no obvious heterogeneous high frequency components were observed farther away from the VT origin (Fig 2B). We analyzed 3335 points in the high voltage regions in the right ventricle (bipolar voltage >1.5mV) and the mean SAFE-T value was 1.43±1.69 Hz·mV. In the area of VT origin, the mean SAFE-T value was 5.49±2.10 Hz·mV (Table 3) (also see histogram in S2 Fig and available dataset in S2 File).

**Table 3:** Substrate characteristics.

Variables	
Total Mapping Points	24718 (353±153)
Total Area (cm^2^)	194.76±44.77
Bipolar voltage (mV)	2.20±0.70
Bipolar low voltage area (cm^2^)	4.59±5.57
Bipolar low voltage area (%)	2.49±2.94
Unipolar voltage (mV)	5.64±1.51
Unipolar low voltage area (cm^2^)	4.41±6.58
Unipolar low voltage area (%)	2.56±4.18
**VT Origins**	
Total RVOT VT origins	84
RVOT septum	55 (65)
RVOT free wall	21 (25)
RV body	8 (10)
Border of LVZ	38 (45)
Inside the LVZ	25 (30)
Area with abnormal potentials (cm^2^), pre-ablation	1.5±0.8
Density of abnormal high frequency fractionated signals (sites/cm^2^), pre-ablation	7.5±2.3
Density of abnormal high frequency fractionated signals (sites/cm^2^), post-ablation	0.7±0.6*
**SAFE-T values**	
VT origins (Hz·mV)	5.49±2.10
Surrounding RV and RVOT myocardium (Hz·mV)	1.49±1.69

* p-value <0.001; There was a significant difference between the density of abnormal high frequency fractionated signals in the VT origins before and after ablation.

Values are in n (%) or mean±SD.

RVOT means right ventricular outflow tract; VT, ventricular tachycardia; RV, right ventricular; LVZ, low voltage zone; SAFE-T, simultaneous amplitude frequency electrogram transformation.

**Fig 3:**
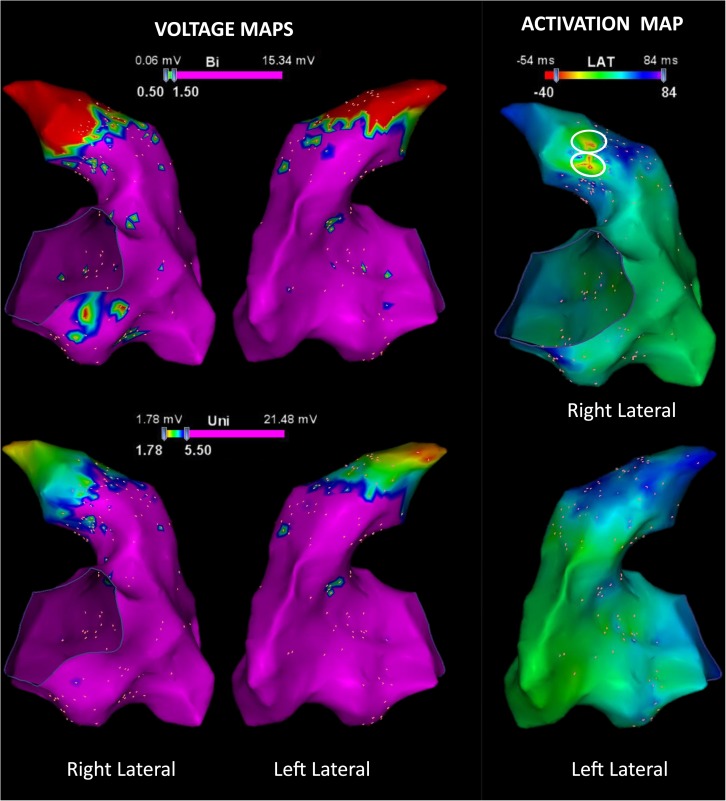
Representative example of the substrate maps during sinus rhythm. (A) Right and left lateral views of the endocardial bipolar and unipolar voltage maps demonstrate the areas of low voltage in the RVOT. (B) The activation map demonstrates the earliest activation site of the RVOT-VT (white circle).

Also, as shown in Table 3, the density of the abnormal high frequency fractionated signals at the VT origin, represented by the SAFE-T maps, was higher compared with other sites in the RVOT and RV body (VT origin, 7.5±2.3 sites/cm^2^ vs. areas outside VT origin, 1.5±1.3 sites/cm^2^, P<0.001). **Fig 3** shows representative example of substrate maps during sinus rhythm displayed on the 3D electroanatomical map. The endocardial bipolar and unipolar voltage maps demonstrated the low voltage areas in the RV outflow tract and RV body. In this study population, 38 (45%) of the VT origins were located at the border of the low voltage zones and 25 (30%) were located within the low voltage area, (low voltage threshold: <1.5 mV in bipolar voltage). There were 21 (25%) VT origins that were located in normal voltage zones.

### Effects of catheter ablation

**Fig 4:**
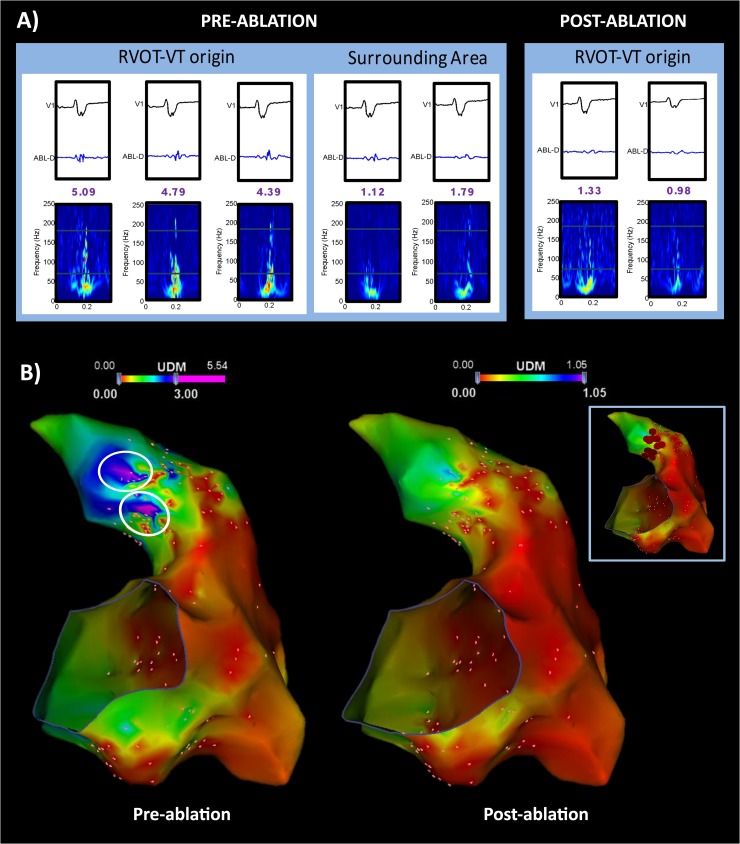
The Hilbert-Huang Transformation (HHT) spectra and SAFE-T maps before and after ablation. (A) The HHT spectra prior to ablation demonstrates high frequency (>70Hz) fractionated potentials at the VT origin with corresponding heterogeneous HHT spectra while the area farther from the origin showed a homogenous HHT spectra with low frequency components (<70Hz). This is displayed in the 3D electroanatomic SAFE-T map (B, left panel) with the VT origins (white circle) showing a SAFE-T value >3.0 Hz·mV. After ablation (A, right panel and B, right panel), the high frequency fractionated potentials were eliminated and the corresponding HHT spectra at the VT origin exhibited a homogenous pattern with reduced high frequency components and SAFE-T value <3.0 Hz·mV.

After the ablation of the VT origins, the areas with high SAFE-T scores were eliminated **(Fig 4**). The density of abnormal high frequency fractionated signals was also significantly decreased after the ablation (after ablation, 0.7±0.6 sites/cm^2^ vs. before ablation, 7.500B12.3 sites/cm^2^, p<0.001).

### Prediction of the VT origin

**Fig 5:**
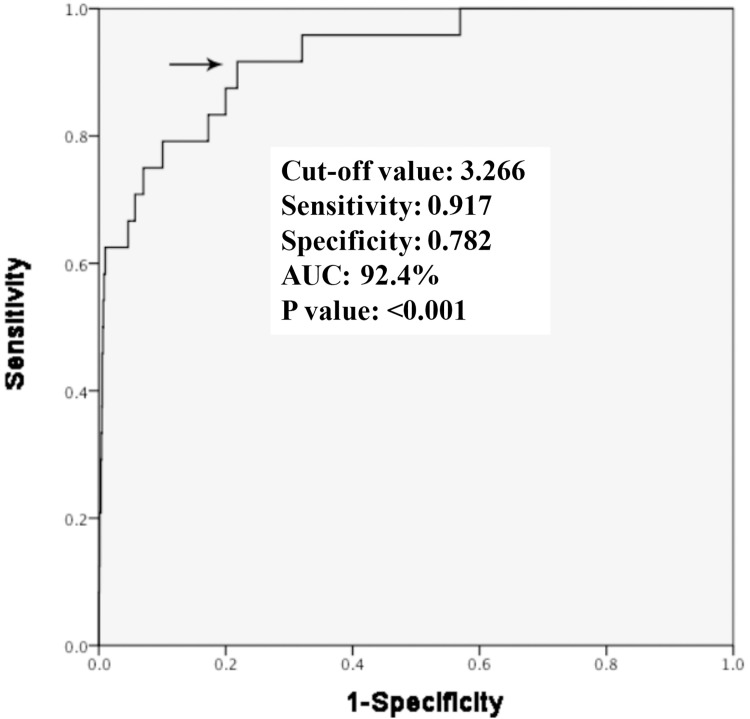
Receiver Operating Characteristic (ROC) curve demonstrates the optimal threshold and goodness of fit for the SAFE-T map for the prediction of focal VT origin.

We performed a receiver operating characteristic (ROC) curve analysis of the generalized estimating equation that exhibited the optimal threshold and goodness of fit for the SAFE-T maps in predicting the focal VT origin with a sensitivity of 91.7%, specificity of 78.2%, cut-off value of 3.266 Hz·mV (**Fig 5**). To simplify, we rounded off the cut-off value for SAFE-T to 3.0 Hz·mV.

## Discussion

### Main findings

We proposed an automated real-time online HHT with a temporal frequency analysis which was used to derived the instantaneous frequency and instantaneous amplitude to calculate for the novel value, SAFE-T value, to characterize the diseased ventricular substrate. This is a novel method that facilitates the identification of the optimal ablation target site in idiopathic RVOT-VTs. We also found a limited region of abnormal potential where the origins of RVOT-VT were successfully ablated. We provided a cut off SAFE-T value for the sites of the VT origin with abnormal high frequency fractionated potentials. To the best of our knowledge, this is the first study to investigate the efficacy of the HHT with a temporal frequency analysis for focal VTs without scar.

### New insights into signal characteristics of VT origins from SAFE-T mapping

The present study used a novel method, the HHT with temporal frequency analysis to derive the instantaneous frequency and instantaneous amplitude in each electrogram and compute for the SAFE-T value, which is the product of instantaneous frequency and amplitude, to quantify the high frequency electrogram during sinus rhythm. This automated online analysis successfully demonstrated that the HHT spectra at the VT origin exhibited a heterogeneous pattern with a high frequency component. On the other hand, there was a homogenous pattern without a high frequency component outside the VT origin. The SAFE-T mapping proposed by this study successfully distinguished the VT origin from other sites. Furthermore, from this retrospective analysis, this novel method can offer an objective way to create an online real-time automated SAFE-T map showing the endocardial distribution of highly fractionated waves. We defined the cut off value for abnormal fractionated electrograms for predicting the VT origin in patients without structurally heart disease at >3 Hz·mV.

### Previous studies of the localization of RVOT-VA origins

An anisotropic conduction pattern around the ventricular outflow tract has been reported to be due to low voltage areas distributed below the pulmonary artery valve and has been suggested to involve preferential conduction from the origin to the breakout site during the tachycardia as shown by high-density mapping.[18,20–23] The earliest activation site of the VT might be identified by local unipolar electrogram with a QS pattern indicating that the vector of the depolarization is spreading away from the earliest activation site toward the surrounding peripheral area.[8] On the contrary, Man et al. suggested that a QS pattern at local unipolar electrogram might not be accurate to identify the successful ablation site.[24] Furthermore, the polarity reversal of bipolar electrogram and negative concordance pattern in the bipolar and unipolar recordings can be useful predictors of the precise localization of the VT origin.[25,26] Although the assessment of the electrogram morphology by the operator’s subjective interpretation might be a feasible strategy for the mapping and ablation of RVOT-VTs, which can be caused by a mechanism of abnormal automaticity or triggered activity,[2,5] and in such a situation, activation mapping might be less helpful due to the transient and/or infrequent appearance of VAs. Recently, Kuteszko et al.[27] reported the efficacy of an automated template matching technique using the surface 12 leads ECG during pace mapping and the clinically documented QRS morphology of VAs. However, previous study by Azegami et al. demonstrated the variable spatial resolution of pace mapping and activation mapping with maximum distance of more than around 20 mm in patients with idiopathic RVOT-VT.[28]

### Previous studies of substrate mapping using a frequency analysis

The fast Fourier transformation (FFT), simplified analysis of Fourier transformation is a mathematical method, which allows visualization of the frequency spectrum of any signal. Pachon et al. reported that a Fourier transform analysis of the bipolar electrogram during sinus rhythm could localize the fibrillar myocardium.[29] They found the FFT spectrum of fibrillar myocardium represents a low power, segmented and heterogenous spectrum due to out of phase conduction property which after ablation showed significant reduction of spectral power with disappearance of high frequency signal. The authors suggest that ablation modifies the heterogeneity of fibrillar myocardium into more homogenous pattern similar to compact myocardium. Our study also demonstrated similar findings that RF ablation abolished or eliminated the fractionated potentials recorded at VT origin corresponding to the high frequency component in the HHT spectra before RF ablation. Recently, Campos et al. reported an automated analysis for quantifying the electrogram fragmentation and evaluated the relationship between the fragmented regions and the VT isthmus in patients with structural heart disease using time-domain and frequency-domain analysis.[30] The authors quantified the area with abnormal high frequency fractionation by determining the ratio of the area under the curve for the high-frequency (>80Hz) to low-frequency (<80Hz) components of the resulting spectra and was calculated as the fast Fourier transform ratio (FFTr). They defined a cut-off value for high FFTr at 14% by examining the FFTr distribution within the bipolar scar area in the post-infarction cardiomyopathy group in their study population.[30] However, the dataset must be linear and strictly periodic or stationary for the proper use of the traditional Fourier spectral analysis.[14] Moreover, in the study by Lin et al.,[15] they demonstrated the utility of the HHT analysis using similar 3D SAFE-T mapping allowing rapid, objective and reliable alternative strategy to late potential mapping in the identification of VT isthmuses in structural heart disease.

In the present study we utilized the same automated electrogram analysis using HHT analysis to derive the SAFE-T value and create the 3D SAFE-T mapping to quantify and characterize the abnormal ventricular electrogram recorded from the VT origins in idiopathic RVOT-VT.

### Clinical implication

This study demonstrated the feasibility of HHT with temporal frequency analysis as an automated real-time/online novel diagnostic tool that could help to localize the VT origins of idiopathic RVOT-VTs. The HHT with a temporal frequency analysis quantified the diseased ventricular substrate in patients without any structural heart disease. We demonstrated that the high frequency fractionated potentials could be displayed as a SAFE-T map combined with the voltage and/or activation maps during the procedure. This provides an objective and quantitative method in identifying the critical site without the induction of VT. To the best of our knowledge, this is the first study to demonstrate the efficacy of the HHT analysis for localizing focal VT origins in those without structural heart disease.

### Study limitations

First, although we developed an automated online signal analysis using the HHT analysis, this was subject in the limitations inherent to a retrospective study design focused only on patients with a successful ablation procedure of the RVOT-VT. Thus, a prospective randomized study to evaluate ablation procedures guided by this novel method applied in real-time mode is warranted. Second, we only created detailed global SAFE-T maps during sinus rhythm or pacing before and after ablation. To avoid any prolongation of the procedure time, we did not confirm the reproducibility of the SAFE-T maps in the same patient in this study. The reproducibility of the novel findings observed from this study should be confirmed.

## Conclusion

This automated online HHT with a temporal frequency analysis was a feasible mapping method for quantifying the diseased ventricular substrate, irrespective of the activation rhythm. A novel automated algorithm proposed by this study could potentially be used to identify the optimal ablation target site for idiopathic RVOT-VTs. Finally, we found a limited region of abnormal potentials in the RVOT-VT origins that were successfully ablated.

## Supporting information

S1 Fig The schematic illustration of the derivation of the Simultaneous Amplitude Frequency Electrogram Transformation (SAFE-T) value from HHT using temporal frequency analysis of normal (left panel) and abnormal (right panel) local bipolar electrograms. (A) shows the ECG (upper panel), local electrogram signal from distal electrode of the ablation catheter (middle panel) and the signal after empirical mode decomposition (EMD) (lower panel). The local electrogram signal (middle panel) is decomposed by EMD into Implicit Mode Functions (IMF) to derived the instantaneous amplitude. (B) The Hilbert transform is applied to each of the IMFs obtained from the EMD to derive the instantaneous frequency. The Hilbert spectrum (HS) is a time-frequency representation of the decomposed signals in A. Inspection of the HS allows identification of the instantaneous frequencies occupying the 70-180Hz band for a significant time duration during each beat. Normal electrograms (left panel) have instantaneous frequencies below the 70Hz band, while abnormal electrograms (right panel) with high frequency fractionated components have instantaneous frequencies within the 70-180Hz band. (C) The simultaneous amplitude frequency electrogram transformation (SAFE-T) value is the product of the instantaneous frequency and instantaneous amplitude derived from the HHT. The normal electrogram (left panel) has low SAFE-T value, while abnormal electrogram (right panel) has a high SAFE-T value (cut-off value >3.0 Hz·mV). SAFE-T readily distinguished the normal from abnormal fractionated electrograms within the high frequency band.
(TIF)

S2 Fig Histogram of the SAFE-T value of local electrograms from the right ventricle. The reference line was set at the cut-off SAFE-T value of 3.0 Hz·mV.
(TIF)

S1 File Derivation of the instantaneous amplitude and frequency from Hilbert-Huang Transform (HHT) for the computation of Simultaneous Amplitude Frequency Electrogram Transformation (SAFE-T) value. (DOCX)

S2 File Data set of the substrate and signal characteristics at the VT origins and surrounding normal myocardium. (PDF)
