# Supplementary figures and images for: The use of a novel signal analysis to identify the origin of idiopathic right ventricular outflow tract ventricular tachycardia during sinus rhythm: Simultaneous amplitude frequency electrogram transformation mapping
Source: PLoS One. 2017 Mar 10;12(3):e0173189. doi: 10.1371/journal.pone.0173189 (PMC5345764; doi:10.1371/journal.pone.0173189)

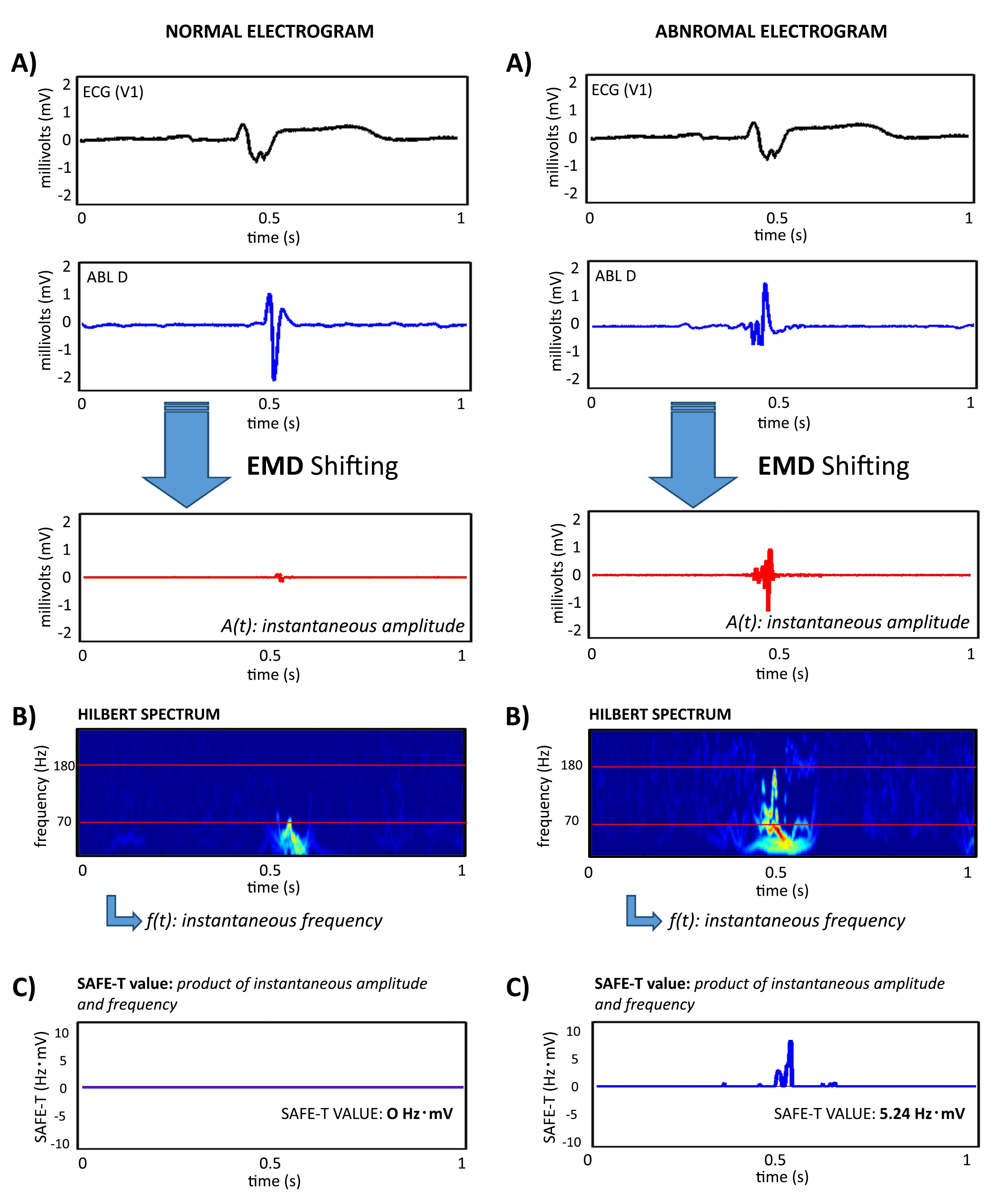

Supplement: S1 Fig — (A) shows the ECG (upper panel), local electrogram signal from distal electrode of the ablation catheter (middle panel) and the signal after empirical mode decomposition (EMD) (lower panel). The local electrogram signal (middle panel) is decomposed by EMD into Implicit Mode Functions (IMF) to derived the instantaneous amplitude. (B) The Hilbert transform is applied to each of the IMFs obtained from the EMD to derive the instantaneous frequency. The Hilbert spectrum (HS) is a time-frequency representation of the decomposed signals in A. Inspection of the HS allows identification of the instantaneous frequencies occupying the 70-180Hz band for a significant time duration during each beat. Normal electrograms (left panel) have instantaneous frequencies below the 70Hz band, while abnormal electrograms (right panel) with high frequency fractionated components have instantaneous frequencies within the 70-180Hz band. (C) The simultaneous amplitude frequency electrogram transformation (SAFE-T) value is the product of the instantaneous frequency and instantaneous amplitude derived from the HHT. The normal electrogram (left panel) has low SAFE-T value, while abnormal electrogram (right panel) has a high SAFE-T value (cut-off value >3.0 Hz·mV). SAFE-T readily distinguished the normal from abnormal fractionated electrograms within the high frequency band. (TIF) [file pone.0173189.s001.tif]

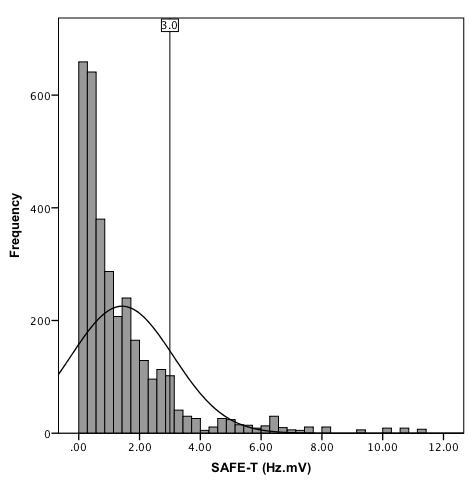

Supplement: S2 Fig — The reference line was set at the cut-off SAFE-T value of 3.0 Hz·mV. (TIF) [file pone.0173189.s002.tif]
