# Supplementary material for: The use of a novel signal analysis to identify the origin of idiopathic right ventricular outflow tract ventricular tachycardia during sinus rhythm: Simultaneous amplitude frequency electrogram transformation mapping
Source: PLoS One. 2017 Mar 10;12(3):e0173189. doi: 10.1371/journal.pone.0173189 (PMC5345764; doi:10.1371/journal.pone.0173189)
Supplement: S1 File — (DOCX) [file pone.0173189.s003.docx]

**Derivation of the instantaneous amplitude and frequency from Hilbert-Huang Transform (HHT) for the computation of Simultaneous Amplitude Frequency Electrogram Transformation (SAFE-T) value**

Recently Huang and its co-workers developed a method, the Hilbert-Huang Transform (HHT), in which the issue of nonlinear and nonstationary data[1] were treated, by using a procedure to decompose the signal according to its own scale properties.[2] HHT was used to extract the periodic components embedded within oscillatory data.[3]

The HHT is comprised of two parts, the empirical mode decomposition (EMD), and the Hilbert Spectral Analysis (HSA). In the first part, the EMD, the kernel of the HHT was used to de-trend a time series and highlight the intrinsic abnormal high frequency potentials embedded within the electrograms. The local electrogram (**S1 FigA**, middle panel) is pre-filtered (at 5-30Hz) prior to EMD. Signal noise is filtered using moving average filter that is built into the system software. The high frequency signals (280 z) and system signals (120Hz, 180Hz) were also filtered prior to EMD. Then, the signal is decomposed by EMD into Implicit Mode Functions (IMF) to derived the instantaneous amplitude, ***A(t)***. So, for a given time series, the sifting procedure of the EMD first identified all local extrema, and then connected all the local maxima and minima by cubic spline lines, defining the upper and lower envelopes. The upper and lower envelopes should have covered all the intervening data. By removing the average of two envelopes, the high frequency components could be sifted into IMFs.

**S1 Fig**. **The schematic illustration of the derivation of the simultaneous amplitude frequency electrogram transformation (SAFE-T) value from HHT using temporal frequency analysis of normal (left panel) and abnormal (right panel) local bipolar electrograms.** (A) shows the ECG (upper panel), local electrogram signal from distal electrode of the ablation catheter (middle panel) and the signal after empirical mode decomposition (EMD) (lower panel). The local electrogram signal (middle panel) is decomposed by EMD into Implicit Mode Functions (IMF) to derived the instantaneous amplitude. (B) The Hilbert transform is applied to each of the IMFs obtained from the EMD to derive the instantaneous frequency. The Hilbert spectrum (HS) is a time-frequency representation of the decomposed signals in A. Inspection of the HS allows identification of the instantaneous frequencies occupying the 70-180Hz band for a significant time duration during each beat. Normal electrograms (left panel) have instantaneous frequencies below the 70Hz band, while abnormal electrograms (right panel) with high frequency fractionated components have instantaneous frequencies within the 70-180Hz band. (C) The simultaneous amplitude frequency electrogram transformation (SAFE-T) value is the product of the instantaneous frequency and instantaneous amplitude derived from the HHT. The normal electrogram (left panel) has low SAFE-T value, while abnormal electrogram (right panel) has a high SAFE-T value (cut-off value >3.0 Hz·mV). SAFE-T readily distinguished the normal from abnormal fractionated electrograms within the high frequency band.

In the second part, we applied the Hilbert transform to the sifted components (IMFs) to derive the instantaneous frequency, ***f(t)***, and constructed its energy-frequency-time distribution from which the time localities of events were preserved (S1 FigB). For any signal *z(t)*, the corresponding analytic signal could be constructed by a combination of the original signal and its Hilbert transform:

*z(t)* = A*(t)*e*^iθ(t)^* (1)

where, $A(t)$ and *θ(t)* are the instantaneous amplitude and instantaneous phase of *z(t)*, respectively.^4^ Instantaneous frequency is defined using the instantaneous variation of phase,


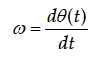


. (2)

This is the second part of the HHT (Hilbert spectral analysis, HAS), and it entails writing the signal in the form


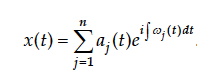


. (3)

Thus, the amplitude and instantaneous frequency as a function of time, H (ϕ,t), can be obtained.^2^ The time frequency representation by the Hilbert spectrum offers a totally different interpretation when compared to the classical Fourier representation. In Fourier terms, the existence of energy at some frequency implies the existence of a component of a sine or cosine wave throughout the whole time-series or a significant part of it. A time-frequency technique allows for the extraction of the frequency content at each time point.^2^ In S1 Fig B, inspection of the Hilbert spectrum allows the identification of the instantaneous frequencies occupying the 70-180Hz band for a significant time duration during each beat. Normal electrograms (S1 Fig B, right panel). Apparently, the local behavior of a signal could be well preserved by the Hilbert Spectrum and was capable of characterizing intermittent, abnormally low-amplitude, high-frequency ventricular activities.

The HHT also enabled the quantification of the local abnormal high frequency components by the product of the instantaneous amplitude and frequency of the electrogram, or the Simultaneous Amplitude Frequency Electrogram Transformation (SAFE-T) value. SAFE-T value could provide a single clinically meaningful parameter in combining both the amplitude and frequency information of the local abnormal bipolar electrograms.

Here, highly fractionated waves usually feature the product over 3 Hz⋅mV. The normal electrograms (S1 Fig C, left panel) has low SAFE-T value, while abnormal electrograms (S1 Fig C, right panel) have high SAFE-T value (cut-off value >3.0 Hz·mV). SAFE-T readily distinguished the normal from abnormal fractionated electrograms within the high frequency band. In addition, an automated time-domain frequency analysis using custom developed software made the analysis procedure rapid and reproducible. The application for SAFE-T mapping for VT in structural heart disease have been previously described in detail.[5]

**References**

1. Huang NE, Wu ZH. A review on Hilbert-Huang Transform: Method and its applications to geophysical studies. Rev. Geophys 2008;46:1-23.
2. Fonseca-Pinto, R. A new tool for nonstationary and nonlinear signals: The Hilbert-Huang Transform in biomedical applications. In: Biomedical Engineering Trends in Electronic Communications and Software, pp. 482–491 (2011).
3. Barnhart, BL. "The Hilbert-Huang Transform: theory, applications, development." PhD (Doctor of Philosophy) thesis, University of Iowa, 2011. http://ir.uiowa.edu/etd/2670.
4. Huang NE, Shen Z, Long SR, Wu MLC, Shih HH, Zheng QN, et al. The empirical mode decomposition and the Hilbert spectrum for nonlinear and non-stationary time series analysis. Proceedings of the Royal Society of London Series A-Mathematical Physical and Engineering Sciences 1998;454:903-995.
5. Lin CY, Silberbauer J, Lin YJ, Lo MT, Lin C, Chang HC, et al. Simultaneous amplitude frequency electrogram transformation (SAFE-T) mapping to identify ventricular tachycardia arrhythmogenic potentials in sinus rhythm. JACC: Clin Electrophysiol 2016;4(2):459-470.
