# Supplementary material for: The use of a novel signal analysis to identify the origin of idiopathic right ventricular outflow tract ventricular tachycardia during sinus rhythm: Simultaneous amplitude frequency electrogram transformation mapping
Source: PLoS One. 2017 Mar 10;12(3):e0173189. doi: 10.1371/journal.pone.0173189 (PMC5345764; doi:10.1371/journal.pone.0173189)
Supplement: S2 File — (PDF) [file pone.0173189.s004.pdf]

Patient No.	Age	Gender	FHx of SCD/VT	SCD Hx	ICD	HTN
1	40	0	0	0	0	1
2	23	0	0	0	0	0
3	32	0	0	0	0	0
4	49	0	0	0	0	0
5	43	1	0	0	0	0
6	35	1	0	0	0	1
7	32	0	0	0	0	0
8	43	1	0	0	0	0
9	34	0	0	0	0	0
10	57	0	0	0	0	1
11	67	0	0	0	0	1
12	31	1	0	0	0	0
13	55	0	0	0	0	0
14	18	1	0	0	0	0
15	38	0	0	0	0	0
16	26	1	0	0	0	0
17	21	0	0	0	0	0
18	39	0	0	0	0	0
19	41	0	0	0	0	0
20	44	0	0	0	0	0
21	36	0	0	0	0	0
22	48	0	0	0	0	1
23	43	0	0	0	0	0
24	43	1	0	0	0	0
25	18	1	0	0	0	0
26	33	0	0	0	0	0
27	38	0	0	0	0	0
28	56	0	0	0	0	0
29	51	1	0	0	0	0
30	37	0	0	0	0	0
31	42	1	0	0	0	0
32	44	0	0	0	0	0
33	58	0	0	0	0	0
34	37	0	0	0	0	0
35	41	0	0	0	0	0
36	38	0	0	0	0	0
37	54	0	0	0	0	0
38	52	0	0	0	0	0
39	35	0	0	0	0	0
40	63	0	0	0	0	0
41	52	1	0	0	0	0
42	32	0	0	0	0	0
43	67	1	0	0	0	0
44	61	0	0	0	0	0
45	45	1	0	0	0	0
46	49	0	0	0	0	0
47	41	0	0	0	0	0
48	37	0	0	0	0	0
49	46	0	0	0	0	0
50	47	0	0	0	0	0
51	36	0	0	0	0	0
52	32	0	0	0	0	0
53	33	0	0	0	0	0
54	24	0	0	0	0	0
55	35	0	0	0	0	0
56	16	1	0	0	0	0
57	45	1	0	0	0	0
58	41	0	0	0	0	0
59	61	0	0	0	0	0

60	26	0	0	0	0	0
61	57	0	0	0	0	1
62	41	1	0	0	0	0
63	62	0	0	0	0	1
64	48	0	0	0	0	0
65	62	0	0	0	0	0
66	44	0	0	0	0	0
67	43	1	0	0	0	0
68	53	1	0	0	0	1
69	54	0	0	0	0	0
70	48	0	0	0	0	0

DM	syncope	palpitation	DOE_SOB	Recurrence During Follow-up	Activation Map	Pacemap
0	1	1	1	0	0	1
0	0	0	0	0	1	0
0	0	1	0	1	1	1
0	0	1	1	0	0	1
0	0	1	0	0	0	1
0	0	1	1	1	0	1
0	0	1	0	0	1	1
0	0	1	0	0	0	1
0	0	1	0	0	0	1
1	0	1	0	0	1	1
0	0	1	0	0	1	1
0	0	0	0	0	1	1
0	0	0	0	0	1	1
0	0	1	0	0	0	1
0	1	1	0	1	0	1
0	0	1	0	0	1	1
0	0	0	0	1	1	1
0	0	1	0	1	0	1
0	0	1	0	1	1	1
0	0	1	0	0	1	1
0	0	1	1	0	1	1
0	0	1	0	0	1	1
0	0	1	1	0	1	1
0	0	1	0	0	0	1
0	0	1	0	1	1	1
0	1	1	1	0	1	1
0	0	1	0	0	1	1
0	0	1	0	0	1	1
0	0	1	0	0	1	1
0	0	1	0	0	1	1
0	0	1	0	0	0	1
0	0	1	1	0	1	1
0	0	0	0	0	1	1
0	0	1	0	0	1	1
0	0	0		0	1	1
0	0	1	0	0	1	1
0	0	1	0	1	1	1
0	0	1	0	0	0	1
0	0	1	0	0	1	1
0	0	0	0	0	0	1
0	0	0	0	0	1	1
0	0	1	0	0	0	1
0	0	1	0	0	1	1
0	0	1	0	0	1	1
0	0	0	0	1	1	1
0	0	1	0	0	1	1
0	0	1	0	0	1	1
0	1	1	0	0	1	1
0	0	1	1	0	1	1
0	0	1	0	0	1	1
0	0	1	0	0	1	1
0	0	1	0	0	0	1
0	0	1	0	0	1	1
0	0	1	0	0	1	1
0	0	1	0	0	0	1
0	0	0	0	0	1	0
0	0	1	0	0	0	1
0	0	1	0	0	1	1
0	0	1	0	0	1	1

0	0	1	0	0	1	1
0	0	1	0	0	1	1
0	0	1	0	0	1	1
1	0	1	0	0	1	1
0	0	1	1	0	1	1
0	0	0	0	0	1	1
0	0	1	0	1	1	1
0	0	0	0	0	1	1
1	0	1	0	0	1	1
0	0	1	0	0	1	1
0	1	1	1	0	1	1

pacemap score	PASO	Earliest activation Time	no. of morphologies	Other arrhythmia	VT origin LVZ border	VT origin inside LVZ	Area of abnormal potential (cm2)
12	98	0	1	0	0	0	0.40
N/A	N/A	34	1	0	1	1	2.80
12	94	36	1	1	0	1	1.50
12	99	0	1	0	1	0	0.50
12	98	97	1	0	1	1	1.30
11	98	32	1	0	0	1	1.20
12	97	21	1	0	0	0	2.60
11	95	0	1	0	0	2	1.00
12	98	0	1	0	1	1	0.90
11	96	52	1	0	1	0	2.80
12	97	38	1	0	0	1	1.10
12	99	58	1	0	1	1	0.50
12	98	42	1	1	1	0	0.60
12	99	0	1	0	0	1	1.80
11	96	0	1	0	0	0	0.70
12	98	38	1	0	1	0	0.40
11	98	40	1	0	1	0	1.60
11	97	0	1	0	1	1	1.10
11	96	33	2	0	2	0	0.90
11	98	46	2	0	0	1	2.40
11	97	39	1	0	0	1	1.40
11	96	35	1	0	0	1	1.60
11	97	30	1	1	0	0	2.40
11	91	0	1	0	0	1	1.50
11	98	38	1	0	1	0	1.60
12	96	42	1	1	1	1	2.30
11	98	40	1	0	1	0	2.30
11	96	36	1	0	0	1	1.50
11	93	45	1	0	0	0	1.10
12	98	31	1	0	0	1	2.30
11	97	0	1	0	1	0	1.10
11	98	40	1	0	0	0	1.60
11	93	51	1	0	0	1	1.40
12	98	50	1	1	1	0	1.70
12	98	40	1	0	1	0	2.80
11	97	38	1	0	0	0	0.90
11	97	41	3	0	2	0	4.00
11	92	0	1	0	1	0	0.70
11	96	51	2	0	1	0	3.10
11	95	34	1	0	0	0	1.90
12	98	52	2	0	1	0	0.70
12	97	0	1	0	1	0	2.30
12	97	42	1	0	0	0	1.20
12	98	22	1	0	1	0	1.10
11	97	52	1	0	0	0	3.30
11	96	45	1	0	1	0	2.00
12	98	40	1	0	0	1	2.10
11	98	44	1	0	0	0	0.40
12	98	36	3	0	1	1	1.90
12	98	36	1	0	1	0	3.00
11	95	32	1	0	0	1	0.70
11	99	0	1	0	1	0	1.10
11	98	38	2	0	0	1	0.00
11	93	51	1	0	0	1	3.90
12	98	0	1	0	0	0	1.10
N/A	N/A	30	1	0	0	0	1.10
11	98	0	1	0	1	0	1.20
12	98	39	1	0	1	0	0.60
11	97	48	1	0	1	0	1.50

11	94	41	1	0	1	0	1.90
12	97	33	1	0	1	0	0.60
11	95	32	1	0	1	0	1.20
11	97	40	1	0	0	0	1.10
12	98	45	2	0	0	0	0.60
11	97	39	1	0	1	0	2.40
11	96	24	1	0	0	0	0.50
12	99	31	1	0	1	1	2.10
11	93	34	1	0	1	0	0.30
12	96	39	1	0	0	0	1.20
12	98	40	1	0	0	0	0.90

Area of abnormal potential (%)	Inducible Sus_VT (EPS)	Inducible NSVT (EPS)	PVC only (EPS)	VPC/VT not induced	RF pulses	Watt	Procedure time
0.20	0	0	0	1	10	60	12.00
1.70	0	0	1	0	22	45	111.00
0.50	0	0	1	0	8	35	32.00
0.20	0	0	0	1	7	40	15.00
0.60	0	0	1	0	18	30	113.00
0.60	0	0	1	0	2	30	32.00
1.80	0	0	0	1	7	30	13.00
0.50	0	0	1	0	10	45	61.00
0.80	0	0	1	0	8	40	11.00
1.30	0	1	0	0	11	45	56.00
0.40	1	1	0	0	9	35	37.20
0.30	0	0	1	0	13	35	53.70
0.30	0	0	1	0	11	35	19.00
0.80	0	0	1	0	7	30	27.00
0.50	0	0	1	0	17	35	89.00
0.30	0	0	1	0	5	35	24.00
1.10	0	1	0	0	20	35	49.00
0.80	0	0	1	0	14	35	20.00
0.70	0	0	1	0	22	35	80.00
0.70	0	0	1	0	26	40	67.00
0.70	0	0	1	0	5	35	28.00
0.90	0	0	1	0	16	35	73.00
2.00	1	0	0	0	8	35	20.00
0.80	0	0	1	0	6	30	25.00
0.80	0	0	1	0	13	30	61.00
1.00	0	1	1	0	27	30	76.00
0.90	0	1	0	0	5	35	17.00
0.80	0	1	0	0	14	35	30.00
0.30	0	1	0	0	7	40	10.00
0.50	1	1	0	0	7	35	24.00
0.40	0	1	0	0	7	30	15.00
0.80	0	1	0	0	9	35	40.00
0.80	0	0	1	0	9	35	17.00
0.90	0	1	0	0	13	30	102.00
1.10	0	0	1	0	7	30	7.00
0.60	0	0	1	0	7	35	40.00
1.90	0	0	1	0	12	35	45.50
0.30	0	0	1	0	9	30	34.00
1.60	0	1	0	0	15	30	33.00
1.00	0	0	1	0	12	35	32.00
0.50	0	1	0	0	23	35	46.00
1.60	0	0	1	0	6	35	12.00
0.60	0	0	1	0	6	40	17.00
0.80	0	0	1	0	7	35	11.00
1.30	0	0	1	0	34	40	65.00
0.90	0	0	1	0	4	35	37.00
1.00	0	0	1	0	8	35	35.00
0.20	0	0	1	0	5	35	21.00
2.70	0	0	1	0	6	35	58.00
1.40	0	1	0	0	10	40	11.50
0.50	0	0	1	0	6	40	18.00
0.70	1	0	0	0	11	35	26.00
0.00	0	0	1	0	15	40	72.80
1.90	0	0	1	0	6	35	48.00
0.60	0	0	1	0	11	35	40.00
0.90	0	0	1	0	11	35	14.00
0.80	0	0	1	0	4	40	7.00
0.30	0	0	1	0	5	35	34.00
0.80	0	0	1	0	5	30	10.00

1.40	0	0	1	0	21	30	54.00
0.30	0	0	1	0	10	35	34.00
0.80	0	0	0	1	13	40	32.00
1.60	0	0	1	0	4	35	16.00
0.30	0	0	1	0	15	35	27.00
1.00	0	0	1	0	19	35	79.00
0.30	0	0	1	0	18	35	59.00
1.40	0	0	1	0	8	35	20.00
0.20	0	0	1	0	4	50	12.00
0.60	0	1	0	0	5	30	20.00
0.40	0	0	1	0	8	35	22.00

Fluoroscoy time	RV_endo	Total mapping points	RV_Uni_Mean_voltage	RV_Bi_Mean_voltage	RV_Total_activation_time	RV_Total_area
10.20	1	329	6.19	3.18	212	218.9
75.00	1	143	3.44	1.88	200	165.6
19.60	1	175	2.64	1.3	174	309.8
4.60	1	555	3.47	1.44	310	192
46.40	1	416	3.69	1.51	360	213.5
21.10	1	378	2.54	2.91	261	197
18.90	1	347	4.16	2	278	145.9
67.00	1	540	6.75	3.03	278	217.7
11.00	1	340	3.9	1.6	145	108.2
36.40	1	299	4.85	1.44	238	213.3
30.70	1	286	4.99	1.66	174	251
46.00	1	346	7.6	1.8	177	167.6
12.80	1	352	6.3	2.2	139	203.9
23.30	1	483	8.9	2.6	157	242
51.50	1	238	6.69	1.64	94	153.5
22.00	1	300	6.92	2.44	77	216.9
39.00	1	244	5.51	1.52	67	152.9
5.40	1	256	4.37	1.58	142	134.8
15.20	1	186	5	1.8	176	153.5
46.00	1	352	5.02	1.8	123	328.9
7.40	1	693	5.7	2	114	191.4
65.00	1	538	5.24	1.7	118	185.9
15.40	1	40	7.14	1.74	132	119.6
14.90	1	396	5.78	1.95	157	188.6
33.90	1	306	6.47	2.45	157	205.8
47.80	1	226	4.52	2.15	130	243
15.60	1	306	5.41	2.56	186	259
10.60	1	152	5.7	2.68	106	192.8
3.90	1	484	6.85	3.14	223	305.9
5.10		255	4.88	1.38	253	160.6
10.40	1	44	6.78	2.07	219	263.8
25.60	1	611	5.32	1.87	194	215.7
12.80	1	463	3.34	1.63	154	169
49.90	1	243	4.45	1.9	156	184.4
3.30	1	669	3.02	1.71	135	242
21.00	1	255	4.34	1.74	96	173.6
35.90	1	358	5.62	2.92	76	216
18.90	1	448	5.98	1.7	158	234.9
13.00	1	265	4.01	1.55	154	190.3
24.70	1	271	5.6	2.26	135	194.3
18.30	1	193	6.79	2.21	105	152
4.20	1	482	4.12	1.19	90	143.4
6.10	1	319	7.41	3.02	93	216
10.30	1	276	4.28	1.62	149	131.9
54.40	1	426	6.27	1.67	115	253.1
6.60	1	391	5.7	1.7	73	209.2
22.00	1	653	5.3	2.02	132	204
7.40	1	617	6.82	2.84	102	190.6
27.80	1	283	7.62	2.8	78	135.5
6.00	1	800	5.01	1.54	90	217.9
10.10	1	296	6.97	2.17	106	145.2
20.00	1	158	7.55	2.72	109	162.6
57.80	1	518	5.58	1.99	105	173.2
36.00	1	384	6.2	2.57	176	202
21.00	1	351	5.69	1.57	126	198.6
9.40	1	78	9.52	3.64	211	248.7
3.30	1	309	3.77	1.56	87	141.8
16.60	1	452	7.8	3.34	126	212.6
6.10	1	478	5.78	2.84	153	190.2

25.90	1	439	6.24	1.7	75	138.4
23.00	1	230	5.56	2.41	83	184
19.10	1	287	9.16	3.68	104	140
3.50	1	309	7.67	3.53	144	151.4
20.20	1	255	8.09	4.15	102	223.4
35.70	1	553	6.58	3.4	103	252.5
20.80	1	379	6.23	2.67	126	178.9
14.50	1	313	4.2	1.6	83	151.4
12.30	1	162	4.49	3.36	103	159.5
5.30	1	293	4.77	1.83	131	200.3
18.70	1	446	4.49	1.3	144	201.1

RV_Uni_LVZ	RV_Uni_LVZ_percentage	RV_Uni_Scar	RV_Uni_sacr_percentage	RV_Bi_LVZ	RV_Bi_LVZ_percentage	RV_Bi_Scar
0	0	0	0	0.4	0.2	0
11.3	6.8	0	0	8.2	4.9	0
0	0	0	0	5.4	1.7	0
21.4	11.14	0	0	24.5	12.7	0
29.5	13.8	0	0	11.7	5.5	0
4	2.03	0	0	2.7	1.4	0
19.1	13.09	0	0	9.7	6.6	0
0	0	0	0	0	0	0
23	21.25	0	0	7.9	7.3	0
0	0	0	0	6.9	3.2	0
0	0	0	0	8.3	3.3	0
5.9	3.5	0	0	5.5	3.3	0
2.2	1.1	0	0	1.6	0.8	0
1.3	0.5	0	0	2	0.8	0
0	0	0	0	0	0	0
1.6	0.7	0	0	2.3	1	0
7.4	4.8	0	0	8.6	5.6	0
8.5	6.3	0	0	6.2	4.7	0
2.5	1.7	0	0	7.9	5.2	0
4.5	1.4	0	0	8.1	2.5	0
10.4	5.4	0	0	10.5	5.7	0
15.1	0.59	0	0	16.2	8.7	0
0	0	0	0	0	0	0
5.4	2.8	0	0	5	2.7	0
0	0	0	0	0	0	0
1.5	0.6	0	0	4	1.6	0
2.9	1.11	0	0	5	1.9	0
0	0	0	0	5.4	2.8	0
0	0	0	0	0	0	0
2.6	1.6	0	0	2.8	1.7	0
2.5	1	0	0	8.6	3.3	0
0	0	0	0	0	0	0
6.7	9.88	0	0	4.6	2.7	0
0	0	0	0	2.2	1.2	0
18	7.4	0	0	27.8	11.5	0
10.5	6	0	0	7.7	4.4	0
0	0	0	0	0	0	0
0	0	0	0	0	0	0
12.7	6.7	0	0	8.5	4.5	0
0	0	0	0	0	0	0
0	0	0	0	0	0	0
0	0	0	0	0	0	0
0	0	0	0	0	0	0
14.2	10.76	0	0	9.2	7	0
0	0	0	0	0	0	0
0	0	0	0	2.8	1.4	0
9.6	4.7	0	0	11.7	5.7	0
0	0	0	0	0	0	0
2.9	2.1	0	0	5.5	4.1	0
0	0	0	0	10.4	4.8	0
0	0	0	0	1.1	0.7	0
0	0	0	0	2.9	1.8	0
5.9	3.4	0	0	13.9	8	3.8
4.9	2.4	0	0	2.2	1.1	0
10	5.1	0	0	0	0	0
0	0	0	0	0	0	0
14	9.9	0	0	10.9	7.7	0
0	0	0	0	0	0	0
0	0	0	0	2.1	1.1	1

6.9	5	0	0	4.4	3.2	0
5.9	3.2	0	0	1.9	1	0
0	0	0	0	0.9	0.6	0
0	0	0	0	0	0	0
0	0	0	0	0	0	0
0	0	0	0	0	0	0
0	0	0	0	0	0	0
0	0	0	0	0	0	0
0	0	0	0	1.5	0.9	0
0	0	0	0	3.7	1.8	0
4.4	1.7	0	0	0	0	0

RV_Bi_Scar_percentage	Density SAFE-T>3.0 (pre-ablation)	Density SAFE-T>3.0 (post-ablation)				
0	9.89	0.300				
0	7.05	0.050				
0	5.88	0.690				
0	4.23	0.290				
0	9.00	0.110				
0	8.76	0.210				
0	8.01	0.230				
0	9.34	0.670				
0	4.34	0.890				
0	4.00	0.770				
0	8.11	0.010				
0	7.68	0.016				
0	9.12	0.761				
0	10.54	0.004				
0	7.46	0.046				
0	6.82	0.234				
0	9.12	0.234				
0	4.44	0.155				
0	6.36	0.132				
0	7.38	1.654				
0	6.23	0.876				
0	6.45	0.825				
0	10.64	1.108				
0	7.00	1.486				
0	2.12	1.396				
0	4.13	1.901				
0	8.12	1.087				
0	10.49	1.234				
0	11.54	1.175				
0	8.00	1.181				
0	6.66	0.000				
0	9.12	1.231				
0	6.45	0.120				
0	5.43	1.089				
0	5.97	1.002				
0	9.18	2.109				
0	5.56	1.760				
0	7.12	1.560				
0	7.90	2.001				
0	8.16	1.924				
0	8.00	1.223				
0	6.00	1.565				
0	9.99	2.156				
0	5.67	0.945				
0	6.14	1.092				
0	6.24	0.678				
0	11.14	1.343				
0	9.02	0.234				
0	8.00	0.142				
0	6.74	0.234				
0	7.00	0.435				
0	8.16	0.123				
2.2	8.14	0.769				
0	7.77	0.222				
0	8.56	0.566				
0	7.72	0.134				
0	8.54	0.222				
0	5.00	0.273				
0.5	8.71	0.543				

0	5.14	0.430				
0	4.13	0.766				
0	5.87	0.345				
0	7.08	0.134				
0	9.13	0.234				
0	7.10	0.234				
0	4.77	0.570				
0	8.67	0.112				
0	16.99	0.123				
0	8.99	0.570				
0	10.76	0.230				

